# Prevalence of Disability among the Chinese Older Population: A Systematic Review and Meta-Analysis

**DOI:** 10.3390/ijerph19031656

**Published:** 2022-01-31

**Authors:** Pian-Pian Zheng, Zi-Le Guo, Xiao-Jing Du, Han-Mo Yang, Zhen-Jie Wang

**Affiliations:** 1 Institute of Population Research, Peking University, Beijing 100871, China; pian714@pku.edu.cn (P.-P.Z.); guozile@stu.pku.edu.cn (Z.-L.G.); 2 School of Humanities and Social Sciences, Xi′an Jiaotong University, Xi′an 710049, China; xiaojingdu@stu.xjtu.edu.cn; 3 National School of Development, Peking University, Beijing 100871, China; hanmo.yang@pku.edu.cn

**Keywords:** prevalence, disability, activities of daily living, older population, Chinese, meta-analysis

## Abstract

Background: Disability is an important problem in aging societies globally. However, the research findings of the prevalence of disability have been inconsistent. This study aims to estimate the prevalence of disability and its influencing factors among the Chinese older population from 1979 to 31 July 2021. Methods: A systematic review and meta-analysis were conducted using both international (PubMed, Web of Science, CBMdisc, PsycINFO, the Cochrane Library, and EMBASE) and Chinese (CNKI, CQVIP, and WanFang) databases. Meta-analysis was performed using a random-effects model to account for heterogeneity. Subgroup analyses were also done. Results: The pooled prevalence of disability across all 97 studies was 26.2% (95% CI: 23.7–28.6%). The estimates varied according to the types of activities of daily living (ADL), gender, age, and region. Studies based on the identification of cases by using the complete ADL scale showed a higher prevalence than those using the basic ADL scale. The prevalence was slightly higher among female older individuals than among male older individuals. The highest rates were seen in older individuals aged 80 years or older. Elders in central China, southwest China, and northwest China were more likely to be BADL-disabled. Conclusion: Prevalence of disability among the Chinese older population is high, around 26%. Using standardized diagnostic systems to correctly estimate the prevalence of disability would be helpful for public health professionals in China.

## 1. Introduction

With the continuous extension of average life expectancy, the proportion of older individuals is increasing dramatically. In 2017, there were an estimated 962 million people aged 60 years or above, accounting for 13% of the global population [1]. The older population in China has reached a prevalence of 18.7%, according to China’s seventh census [2]. With the rapid aging that is occurring in all regions of the world, the prevalence of older individuals in the whole world, except Africa, will reach 25% by 2050. Therefore, the problems associated with an aging society are becoming more severe, and one of the associated problems is a high rate of disability. Figuring out the rate of disability and the number of disabled elders is vital for a country to make improvements and promotion strategies for the old, disabled population’s quality of life. Relevant agencies can also make better plans for financial support, nursing services, and medical services.

According to the World Health Organization (WHO) report on disability, the estimated prevalence of disability was 10.2% in people aged 60 years or above in 194 countries and regions around the world [3]. However, in China, the second national sample survey on disability pointed out that the disabled elders accounted for 24.43% of all older individuals [4]. This difference in the prevalence of disability was more apparent in individual studies. We found that the prevalence of disability among the Chinese older population varies greatly, ranging from 1.85% to 71.28% [5,6], after combining the results of different studies. The pooled prevalence rates given by three relevant meta-analyses were 20.1% (95% CI: 14.7–25.6%), 28.5% (95% CI: 25.9–31.2%), and 34% (95% CI: 14–53%), which also seem to be quite different [7,8,9].

One of the reasons for this discrepancy is the different understanding and measurements. Disability can be defined in various ways, including impairment, limitations in mobility, physical function decline, and activities of daily living (ADLs). World Health Organization (WHO)’s International Classification of Functioning, Disability, and Health (ICF) uses disability as an umbrella term for impairments, activity limitations, or participation restrictions [10]. Additionally, it points out that disability represents the negative aspects of the interaction between the health condition and life situations (personal factors and environmental factors). Thus, the ADL is considered a suitable measurement of disability and also has good robustness and comparability [11,12].

Although several studies calculated the prevalence of ADL disability among the older population, a synthesis of these studies to derive a general risk estimate has not been well conducted. Hence, we have carried out a systematic review and meta-analysis to comprehensively analyze the related studies and extract a more accurate and general prevalence of disability by avoiding differences in individual studies caused by biased samples and moderating factors.

## 2. Materials and Methods

### 2.1. Literature Search

This research protocol was registered with the International Prospective Register of Systematic Reviews (PROSPERO: CRD42021269367). The literature search was conducted using the Chinese National Knowledge Infrastructure (CNKI), VIP Database (CQVIP), China Biology Medicine disc (CBMdisc), Wanfang, PubMed, Web of Science, Embase, and the Cochrane Library. All databases were searched from 1979 (the earliest year available on the CNKI database) to 31 July 2021. The search terms were keywords related to the older population (elderly OR elder OR old population OR old adults), ADL (activities of daily living OR disable OR disabled OR ADL OR BADL OR IADL), and China (China OR Chinese).

The screening procedure is shown below: (a) the titles were reviewed to determine potential articles related to the topic, (b) the abstracts were reviewed to narrow down the list of articles, and (c) the full text of the articles was read to make a final decision.

### 2.2. Inclusion and Exclusion Criteria

The complete type of ADL includes basic activities of daily living (BADL) and instrumental activities of daily living (IADL). The most commonly used scales for assessing BADL are the Katz independence index and the Barthel independence index, which have 6 or 10 items, consisting of relatively simple self-care tasks such as dressing, eating, and bathing [13,14]. Moreover, the frequently used tool for assessing IADL was designed by Lawton and Brody and has 8 items, such as shopping, cooking, financial management, and other more complex activities [15]. Those standard scales provide good support for us to merge the rates and compare. Some researchers used a self-made scale or increased or decreased the ADL items, and we did not include these studies.

Studies were included only if they met the following criteria: (a) the study was published between 1979 and 31 July 2021; (b) the study was conducted using a questionnaire survey, and the measurement tool was the Barthel independence index, the Katz independence index or the scale designed by Lawton and Brody; (c) the study reported the prevalence of disability with accurate and clear data; and (d) all older respondents were aged 60 years or above and came from Mainland China.

Studies were excluded if they met the following criteria: (a) for literature published with the same data, only the latest data were included; (b) reviews, conferences, lectures, or unpublished essays; (b) an unscientific research design, such as convenience sampling, was used in the study; and (c) the study was based on a sample population involving patients, elders living in nursing homes, and other special groups with specific health-related characteristics.

### 2.3. Data Extraction

The data in the studies, including the authors, publication year, survey year, sampling locations, diagnostic tools, participants, and disability cases, were collected. Additionally, the prevalence of disability among older individuals of different diagnostic tools, genders, ages, and regions was collected.

All studies were reviewed and coded by two authors to determine the consistency of the inclusion and exclusion criteria. In addition, each study included in the meta-analysis was coded by two authors to extract major outcomes. The discrepancies were resolved through discussions.

### 2.4. Quality of Assessment

The quality of included studies was assessed by the 11-item checklist recommended by the Agency for Healthcare Research and Quality (AHRQ). The item would be scored 1 for the answer of “Yes” and would get a score of 0 if the answer was “No” or “Unclear” (opposite for the 5th item). A total score of 0–3 = low quality, 4–7 = moderate quality, and 8–11 = high quality [16].

### 2.5. Statistical Analysis

The meta-analysis was carried out by using STATA 16.0. Combined effect sizes with corresponding confidence intervals (95%) were calculated, and these indicated the magnitude of the effect across all studies. The Q test and I^2^ statistics were used to assess heterogeneity among the included studies. *p* > 0.05 and I^2^ < 50% indicated no statistical heterogeneity between the studies. If no heterogeneity was observed, the fixed-effects model was employed; otherwise, the random-effects model was used [17]. The homogeneity test showed that Q = 81,405.53 (*p* < 0.001) and I^2^ = 99.9%. Therefore, we adopted the random-effects model for all meta-analyses.

Subgroup analyses and meta-regression analyses were conducted to eliminate heterogeneity and identify potential influencing factors. Sensitivity analyses were conducted by removing one study at a time and then recalculating the prevalence of the remaining studies to test the robustness of the primary results. Publication bias was diagnosed through Begg’s test. The significance level was set at 0.05 (two-sided) in all analyses.

## 3. Results

### 3.1. Search Strategy and Selection Criteria

**Figure 1:**
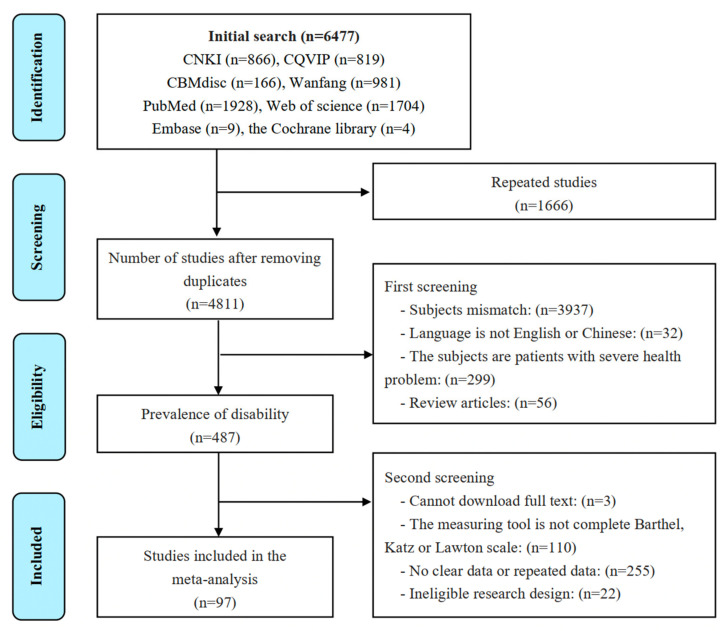
Flow chart of the study selection process.

Figure 1 shows a flow diagram of the systematic search of the literature. A total of 6444 articles were identified in 8 electronic databases. Among them, 1666 duplicates were eliminated, the titles and abstracts studies were screened, and the full text of 484 studies was evaluated. In the end, 97 studies passed the evaluation and were included in the meta-analysis.

### 3.2. Quality Assessment

The results of the quality assessment are shown in Table 1. Based on the AHRQ checklist, 97 studies reached moderate quality and above.

### 3.3. Study Characteristics

Table A1 in Appendix A summarizes the characteristics and findings of the included studies. A total of 97 eligible studies reported the prevalence of disability in Chinese older individuals, with a total of 110 results. Eight studies reported multiple results because they used several cross-sectional data sets or used several types of ADL.

Most of the included studies were cross-sectional, and two were longitudinal. For the longitudinal study, we included only the results from the cross-sectional analysis of the baseline data. In addition, 18 studies used national data, while the remaining studies obtained samples from regions within China; 86 studies were conducted with the general older population (≥60 or ≥65 years), but 11 studies only included the oldest elders (≥80 years) or centenarians. The sample size ranged from 182 to 32,281. The time of data collection spanned nearly three decades.

### 3.4. Pooled Prevalence of Disability

In total, 97 studies met the inclusion criteria, with 110 results. The whole sample included 561,800 subjects, of whom 116,813 had disabilities. Table 2 shows that the pooled prevalence of disability among the Chinese older population was 26.2% (95% CI: 23.7–28.6%).

### 3.5. Subgroup Analyses

The prevalence varied greatly according to the types of ADL. The prevalence of disability detected by BADL was 20.5% (95% CI: 17.7–23.3%), which was significantly lower than that detected by complete ADL (33.8%, 95% CI: 29.4–38.3%) (*p* < 0.001).

The prevalence in women (28.5%, 95% CI: 24.5–32.5%) was slightly higher than that in men (22.7%, 95% CI: 20.0–25.5%). A significant difference was found among different age groups (*p* < 0.001). The prevalence of disability in the oldest age group (≥80 years) was 36.8% (95% CI: 33.1–40.5%), which was higher than that in the 60–69 years age group (12.8%, 95% CI: 10.1–15.5%) and the 70–80 years age group (22.4%, 95% CI: 16.5–28.3%).

### 3.6. Assessment of Disability by Using a Specific Type of ADL

#### 3.6.1. BADL

As Table 3 shown, 56 studies provided information about the BADL. The random-effects analysis showed that the pooled prevalence of BADL disability was 20.5% (95% CI: 17.7–23.3%). Furthermore, older individuals aged 80 years or over (30.0%, 95% CI: 26.2–33.9%, *p* < 0.001) had a significantly higher BADL disability rate than younger elders. To avoid the limitation of insufficient studies, we merged some regional subgroups and found that other parts of China had an obviously higher BADL prevalence (24.4%, 95% CI: 26.2–33.9%, *p* < 0.001) than northern China.

#### 3.6.2. Complete ADL

As Table 4 shown, 41 studies combined the basic and instrumental activities of daily living as a complete measurement tool, which consisted of 14 items. The pooled prevalence of disability according to complete ADL was 33.8% (95% CI: 29.4–38.3%). The oldest elders (≥80 years) also had an evidently higher prevalence (61.9%, 95% CI: 51.9–71.9%, *p* < 0.001).

### 3.7. Meta-Regression

In this study, score, mean age, the proportion of females, the proportion of rural hukou, publication year, and survey year can be taken as a continuous variable. Additionally, meta-regression was performed to assess the relationship between those variables and the pooled prevalence. The results showed that only mean age had a significant linear relationship with the prevalence of disability (b = 0.0094, *p* < 0.001). Thus, the prevalence of ADL disability in Chinese older adults showed an ascending trend with age.

### 3.8. Publication Bias and Sensitivity Analyses

Begg’s test showed that there was no obvious publication bias (z = 1.65, *p* = 0.099). The results of sensitivity analysis were between 25.7% (95% CI: 23.3–28.2%) and 26.4% (95% CI: 23.9–28.9%), indicating that the primary result had good robustness.

## 4. Discussion

To the best of our knowledge, this is the first meta-analysis to examine the prevalence of disability among older adults in mainland China over such an extensive period based on both international and Chinese databases. The total number of older persons in this analysis was large enough to be conclusive on several issues. The meta-analysis of 97 studies revealed that the prevalence was 26.2% (95% CI: 23.7–28.6%), which means there are nearly 69 million older people suffering from disabilities in China. Additionally, the prevalence of disability presented differences in terms of types of ADL, gender, age, and region.

We divided ADL into three types when collating and analyzing the data and found that we obtained a higher pooled prevalence for complete ADL, especially compared with BADL. These findings might be related to the characteristics of BADL and IADL. BADL and IADL represent different positions along the spectrum of the disablement process. BADL reflects the elders’ basic self-care independence, whereas IADL reflects the ability of older people to live independently. The IADL disability is more likely to happen earlier with age. Hence, the more items that are included, the more sensitive the tool will be.

Several studies had shown that the ADL ability of older individuals was negatively correlated with age [18,19]. Therefore, it is not surprising that the pooled prevalence of disability among the oldest people (age 80 years or above) was significantly higher than that of younger individuals. Meanwhile, the regression results suggest that the increase in the prevalence of disability is about a 0.09 percent point for each 1-year increase in the mean age of the population. With increasing age, the physiological functions of older adults continue to decline, the risk of chronic disease and accidental injury increases, and the disability trend could further increase [20].

The prevalence of disability differs significantly by gender, and the prevalence in females was significantly higher than that in males. This finding is consistent with the results of numerous studies, showing that females are more likely to experience disabilities [21]. Especially in BADL disability, that females’ disability rate was 1.29 times that of males. This difference was mainly attributed to two aspects. Compared with females, males have usually had better social status, income, and degree of education since ancient times. They have a stronger awareness of health care and more social resources to obtain health care [22]. In addition, the average life expectancy of females is longer than that of males [23], leading to a higher risk of disability.

Compared with northern China, elders in other regions (including central China, southwest China, and northwest China) were more likely to be BADL-disabled. Although the economic conditions have been greatly improved recently in most areas of China, many older adults living in remote areas are still unable to obtain timely and high-quality medical services.

This analysis provides useful information for the public health professionals of China. Over a quarter of all Chinese older individuals may have different levels of disability. This result indicates that we should strengthen community-based intervention and provide more health services, such as disability assessments and functional exercises. Once an individual has a severe functional impairment, medical assistance and financial subsidies should be provided promptly. This is especially true for older individuals aged over 80 years and female older individuals.

## 5. Limitations

There are some limitations of our study. First, this study included only published studies, and there may have been publication bias even though no such bias was indicated by statistical tests. Second, the included studies suffered from high heterogeneity, although the measurement tools were controlled and subgroup analyses were performed to address this shortcoming. High heterogeneity may reflect differences in the design and conduct of the studies (methodological heterogeneity) or in the participants and outcomes measured (clinical heterogeneity) [24,25]. In this meta-analysis, we collected many studies published over nearly three decades. It was inevitable that we did not fully identify the studies with low-quality research design. Additionally, we may have ignored some important confounding factors, such as disease and social-economic status. Moreover, the large sample size included in the study may also make the I^2^ value increase [26]. In addition, when using dependency measures, much information about the severity of the disability was lost, which is worthy of further study.

## 6. Conclusions

The meta-analysis of 97 studies on the prevalence of disability among the Chinese elderly population from 1979 to 2021 found that (1) the pooled prevalence reached 26.2% (95% CI: 23.7–28.6%) and (2) differences in prevalence exist in terms of types of ADL, gender and age. Considering the negative impact of disability on personal well-being and financial expenditure, regular and appropriate interventions are needed for this vulnerable group.

## Figures and Tables

**Table 1:** Risk of bias using quality assessment forms.

Item	Yes	No	Unclear
(1) Define the source of information (survey, record review)	97	0	0
(2) List inclusion and exclusion criteria for exposed and unexposed subjects (cases and controls) or refer to previous publications	97	0	0
(3) Indicate time period used for identifying patients	78	19	0
(4) Indicate whether or not subjects were consecutive if not population-based	97	0	0
(5) Indicate if evaluators of subjective components of the study were masked to other aspects of the status of the participants	0	97	0
(6) Describe any assessments undertaken for quality assurance purposes (e.g., test/retest of primary outcome measurements)	60	36	1
(7) Explain any patient exclusions from the analysis	89	7	1
(8) Describe how confounding was assessed and/or controlled.	65	32	0
(9) If applicable, explain how missing data were handled in the analysis	13	82	2
(10) Summarize patient response rates and completeness of data collection	86	11	0
(11) Clarify what follow-up, if any, was expected and the percentage of patients for which incomplete data or follow-up was obtained	0	97	0

**Table 2:** Pooled prevalence of disability and subgroup analyses.

Variables	Classification	Number of Studies	Number of Results	Event Rate (%)	95% CI (%)	Heterogeneity I^2^ (%)	Q-Value	*p*-Value
Pooled prevalence		97	110	26.2	23.7–28.6	99.9	81,405.53	
Type of ADL	BADL	56	62	20.5	17.7–23.3	99.9	26.55	<0.001
	IADL	7	7	31.8	21.2–42.4	99.9		
	BADL + IADL	41	41	33.8	29.4–38.3	99.6		
Gender	Male	53	60	22.7	20.0–25.5	99.7	5.35	0.021
	Female	53	60	28.5	24.5–32.5	99.8		
Age group	60–69	23	26	12.8	10.1–15.5	99.6	104.92	<0.001
	70–79	23	26	22.4	16.5–28.3	99.7		
	≥80	36	44	36.8	33.1–40.5	99.6		
Region	Eastern China	32	33	27.0	22.3–31.7	99.8	2.44	0.786
	Northern China	18	20	26.0	19.9–32.1	99.7		
	Southern China	6	6	24.2	8.0–40.3	99.7		
	Central China	7	7	26.9	17.9–35.8	99.4		
	Southwest China	10	13	30.9	22.3–39.4	99.7		
	Northwest China	4	4	21.3	12.3–30.3	97.8		
Hukou	Urban	17	22	22.4	16.9–27.9	99.9	2.13	0.143
	Rural	26	31	28.0	22.9–33.0	99.9		
Survey year	1999 and before	5	6	21.4	10.4–32.4	99.8	2.16	0.706
	2000–2004	6	7	23.7	13.0–34.3	99.8		
	2005–2009	10	12	29.1	21.6–36.7	99.7		
	2010–2014	41	43	27.7	23.6–31.8	99.8		
	2015–2019	36	38	25.3	20.9–29.7	99.9		

**Table 3:** Pooled prevalence of BADL disabilities and subgroup analyses.

Variables	Classification	Number of Studies	Number of Results	Event Rate (%)	95% CI (%)	Heterogeneity I^2^ (%)	Q-Value	*p*-Value
Pooled prevalence		56	62	20.5	17.7–23.3	99.9	45,852.90	
Gender	Male	37	41	19.4	16.4–22.4	99.7	3.95	0.047
	Female	37	41	25.1	20.3–29.9	99.8		
Age group	60–69	17	17	7.3	5.7–8.9	98.6	111.60	<0.001
	70–79	17	17	13.1	10.4–15.9	98.4		
	≥80	29	33	30.0	26.2–33.9	99.6		
Region	Eastern China	15	15	16.8	13.5–20.1	99.3	10.45	0.005
	Northern China	6	8	12.9	9.7–16.1	99.2		
	Other regions *	16	16	24.4	18.0–30.7	99.5		
Hukou	Urban	14	18	22.6	16.1–29.2	99.9	0.00	0.944
	Rural	16	20	22.4	17.4–27.3	99.9		
Survey year	2009 and before *	8	14	21.7	14.2–29.1	99.9	0.78	0.678
	2010–2014	25	25	21.3	17.6–25.1	99.7		
	2015–2019	20	20	18.9	14.3–23.4	99.8		

* To avoid the limitation of insufficient studies, we merged Central China, Southwest China, and Northwest China into a group called “Other regions”. In addition, the studies published in 2009 and before were merged into one group.

**Table 4:** Pooled prevalence of complete ADL and subgroup analyses.

Variables	Classification	Number of Studies	Number of Results	Event Rate (%)	95% CI (%)	Heterogeneity I^2^ (%)	Q-Value	*p*-Value
Pooled prevalence		41	41	33.8	29.4–38.3	99.6	10,997.47	
Gender	Male	16	16	32.2	23.1–41.4	99.5	0.52	0.472
	Female	16	16	36.7	28.7–44.7	99.4		
Age group	60–69	6	6	25.5	14.0–36.9	99.7	22.45	<0.001
	70–79	6	6	40.5	24.9–56.1	99.6		
	≥80	7	7	61.9	51.9–71.9	97.3		
Region	Eastern China	16	16	36.4	27.8–44.9	99.8	1.10	0.578
	Northern China	12	12	34.7	26.9–42.4	99.6		
	Other regions *	12	12	31.2	25.1–37.2	99.6		
Survey year	2009 and before *	10	10	32.9	24.4–41.5	99.5	0.38	0.827
	2010–2014	15	15	35.9	27.4–44.3	99.7		
	2015–2019	16	16	32.5	24.9–40.1	99.7		

* To avoid the limitation of insufficient studies, we merged Central China, Southwest China, and Northwest China into a group called “Other regions”. In addition, the studies published in 2009 and prior to 2009 were merged into one group.

## Data Availability

All relevant data are within the paper.

## Appendix A

**Table A1:** Characteristics of the 97 studies included in the meta-analysis.

NO.	Study	Publication Year	Language	Survey Year	Sampling Province	Age (Mean)	Type of ADL	Sample Size	Female (N, %)	Rural (N, %)	Cases of Disability	Rate	Score of Quality
1.	Huang et al. [27]	1993	CH	1991 *	Sichuan	≥60 (68.8)	PADL + IADL **	1242	657 (52.9)	NR	422	33.98	6
2.	Meng et al. [28]	1996	CH	1992	Beijing	≥60	PADL + IADL	3257	1415 (43.44)	NR	778	23.90	7
3.	Tang et al. [29]	1999	EN	1990	Beijing	≥60 (71.0)	PADL **	3440	1733 (50.38)	NR	629	18.28	9
4.	Lv et al. [30]	2001	CH	2000	Anhui	≥60	PADL + IADL	1424	NR	NR	274	19.24	5
5a.	Meng et al. [31]	2002	CH	1992	Beijing	≥60 (72.3)	PADL	2783	NR	NR	262	9.41	8
5b.	Meng et al. [31]	2002	CH	1997	Beijing	≥60 (72.0)	PADL	2786	NR	NR	171	6.14	8
5c.	Meng et al. [31]	2002	CH	2000	Beijing	≥60 (72.9)	PADL	2667	NR	NR	214	8.02	8
6.	Wang et al. [32]	2002	CH	2000	Guangzhou	≥60	PADL **	1161	631 (54.35)	NR	94	8.10	7
7.	Lin et al. [33]	2002	CH	2000	Beijing	≥60	PADL	895	NR	NR	174	19.44	7
8.	Ji et al. [34]	2007	CH	2005 *	Jiangsu	≥60	PADL + IADL	337	NR	NR	103	30.56	6
9.	Yin and Lu [35]	2007	EN	2002	National	≥80	PADL	8844	4938 (55.83)	4627 (52.3)	3153	35.65	10
10a.	Huang et al. [36]	2008	CH	2006 *	Guizhou	≥60 (70.2)	PADL	3221	1995 (61.94)	NR	171	5.31	7
10b.	Huang et al. [36]	2008	CH	2006 *	Guizhou	≥60 (70.2)	IADL	3221	1995 (61.94)	NR	382	11.86	7
11.	Tang et al. [37]	2009	CH	2008	Hunan	≥60	PADL + IADL	203	124 (61.08)	NR	102	50.25	7
12.	Xu et al. [38]	2011	CH	2009 *	Zhejiang	≥60 (70.0)	PADL + IADL	753	404 (53.65)	753(100.00)	129	17.13	7
13.	Chen et al. [39]	2011	CH	2010	Zhejiang	≥80 (84.8)	PADL **	454	268 (59.03)	NR	138	30.40	9
14.	Li et al. [40]	2011	EN	2006	Beijing	≥60	PADL + IADL	1882	990 (52.60)	NR	817	43.41	5
15.	Xue et al. [41]	2011	CH	2010	Shanghai	≥80 (83.1)	PADL + IADL	1027	140 (13.63)	NR	674	65.63	9
16.	Li et al. [42]	2012	CH	2009	Shanghai	≥60 (73.3)	PADL + IADL	11,338	6043 (53.30)	NR	2013	17.75	8
17.	Shi et al. [43]	2012	CH	2011	Shandong	≥65	PADL	504	234 (46.43)	NR	96	19.05	8
18.	Li et al. [44]	2012	CH	2010 *	Ningxia	≥60	PADL + IADL **	904	459 (50.77)	NR	261	28.87	7
19.	Zhang et al. [45]	2012	CH	2010 *	Hebei	≥60	PADL + IADL **	2161	NR	NR	796	36.83	7
20.	Yu et al. [46]	2012	CH	2011	Shanghai	≥60	PADL + IADL	1500	842 (56.13)	NR	589	39.27	8
21.	Huang et al. [47]	2012	CH	2008	Anhui	≥60 (70.2)	PADL + IADL **	1117	764 (68.40)	1117(100.00)	764	68.40	8
22.	Yin et al. [48]	2012	CH	2009	Zhejiang	≥60 (71.2)	PADL + IADL **	2184	1218 (55.77)	2184(100.00)	566	25.92	8
23.	Zhang and Wei [49]	2014	CH	2013	Beijing	≥60	PADL	2031	NR	NR	200	9.85	9
24.	Zhong et al. [50]	2014	CH	2008	Zhejiang, Gansu	≥60	PADL **	1157	547 (47.28)	647 (55.92)	214	18.50	9
25.	Yin et al. [51]	2014	EN	2011	National	≥80 (92.3)	PADL	5495	3192 (58.09)	NR	1856	33.78	9
26.	Chen et al. [52]	2015	CH	2013*	Fujian	≥60 (71.5)	PADL	14,292	7404 (51.81)	NR	610	4.27	8
27.	Li et al. [53]	2015	CH	2013*	Ningxia	≥60 (70.0)	PADL + IADL **	817	457 (55.94)	NR	84	10.28	7
28.	Li and Yuan [54]	2015	CH	2013	Shandong	≥60	PADL	416	276 (66.19)	172 (41.25)	67	16.11	7
29.	Zhang et al. [55]	2015	CH	2011	Chongqing	≥80	PADL **	227	131 (57.71)	NR	84	37.00	9
30.	Zhang et al. [56]	2016	EN	2013	Shanghai	≥60 (72.1)	IADL	8237	4473 (53.26)	NR	1360	16.51	7
31.	Gong [57]	2016	CH	2014	Shanghai	≥60	PADL + IADL	1233	NR	NR	226	18.33	6
32.	Zhong [58]	2016	CH	2012–2014	Guangdong	≥60	PADL	1706	NR	NR	331	19.40	7
33.	Liu et al. [59]	2016	EN	2013	Beijing	≥60 (71.4)	PADL	1036	522 (50.40)	NR	219	21.10	7
34.	Peng and Wu [60]	2016	CH	2011	National	≥65	PADL	9097	4918 (54.06)	4755 (52.27)	1948	21.41	10
35.	Huang et al. [61]	2016	CH	2013–2015	Zhejiang	≥60 (73.8)	PADL	883	490 (55.49)	NR	191	21.63	8
36a	Su et al. [62]	2016	EN	2013	Shanghai	≥80	PADL	2058	1191 (57.87)	NR	478	23.23	7
36b.	Su et al. [62]	2016	EN	2013	Shanghai	≥80	IADL	2058	1191 (57.87)	NR	780	37.90	7
37.	Yue and Liu [63]	2016	CH	2011	National	≥65	PADL	5118	2861 (55.90)	NR	1214	23.72	10
38.	Chen et al. [64]	2016	CH	2014	Shanghai	≥60 (74.2)	PADL + IADL	3556	2114 (59.45)	NR	879	24.72	8
39.	Yi et al. [65]	2016	CH	2013	Hubei	≥65 (73.3)	PADL + IADL **	4002	2058 (51.42)	4002(100.00)	1375	34.36	8
40.	Zhang et al. [66]	2016	CH	2014*	Hebei	≥60 (68.7)	PADL + IADL **	2548	1322 (51.88)	1350 (52.98)	1076	42.23	7
41.	Zhai et al. [67]	2016	CH	2011	Shandong	≥65	PADL + IADL	1355	706 (52.10)	729 (53.80)	921	67.97	10
42.	Luo et al. [68]	2016	CH	2011	Shandong, Henan, Hebei, Hunan, Guangdong, Guangxi, Hainan, Jiangsu	≥65	PADL **	2227	1227 (55.10)	NR	553	24.83	10
43.	Dong et al. [5]	2017	EN	2011	Shanghai	≥60 (71.6)	PADL	1997	1153 (57.74)	NR	37	1.85	6
44a.	Zhang et al. [69]	2017	EN	2005–2014	National	≥65 (72.0)	PADL	26,604	13,515 (50.80)	16,022 (60.22)	1862	7.00	9
44b.	Zhang et al. [69]	2017	EN	2005–2014	National	≥65 (72.0)	IADL	26,604	13,515 (50.80)	16,022 (60.22)	8513	32.00	9
45.	Zhou and Ma [70]	2017	CH	2013	National	≥60 (68.9)	PADL	7629	3988 (52.27)	7629 (100.00)	668	8.76	9
46.	Ding and Wang [71]	2017	CH	2014	National	60–79 (67.7)	PADL + IADL	6959	3549 (50.99)	3897 (56.00)	1038	14.92	9
47.	Jin [72]	2017	CH	2011	National	≥60	PADL **	9765	NR	NR	2084	21.34	9
48.	Li et al. [73]	2017	CH	2013	Anhui	≥60 (72.3)	PADL + IADL **	746	438 (58.71)	NR	211	28.28	9
49.	Hao et al. [74]	2017	EN	2016	Beijing	≥60	PADL + IADL	1083	543 (50.14)	NR	347	32.04	8
50.	Liu et al. [75]	2017	CH	2016	Shandong	≥65	PADL **	1196	NR	NR	404	33.78	8
51.	Wang et al. [76]	2017	CH	2015*	Hebei	≥60 (75.5)	PADL + IADL **	724	378 (52.20)	NR	309	42.68	6
52a.	Hu et al. [77]	2017	CH	2014	National	≥65 (66.4)	PADL **	6168	2813 (45.61)	NR	1517	24.59	8
52b.	Hu et al. [77]	2017	CH	2014	National	≥65 (66.4)	IADL **	6168	NR	NR	3864	62.65	8
53.	Wu et al. [78]	2017	EN	2010	Chongqing	≥100	PADL	564	471 (83.51)	564 (100.00)	370	65.60	9
54.	Yang et al. [79]	2018	EN	2015–2016	Hubei	≥65 (72.6)	PADL **	2096	1065 (50.81)	NR	149	7.11	8
55.	Liu et al. [80]	2018	CH	2013	National	≥60	PADL **	8751	NR	NR	842	9.62	8
56.	Ding and Yan [81]	2018	CH	2011	National	≥60	PADL	7626	3801 (49.84)	5765 (75.60)	845	11.08	8
57a.	Chen et al. [82]	2018	EN	2016–2017	Guangxi	≥60	PADL	2300	1350 (58.70)	NR	266	11.57	7
57b.	Chen et al. [82]	2018	EN	2016–2017	Guangxi	≥60	IADL	2300	1350 (58.70)	NR	976	42.43	7
57c.	Chen et al. [82]	2018	EN	2016–2017	Guangxi	≥60	PADL + IADL	2300	1350 (58.70)	NR	998	43.39	7
58.	Zhai et al. [83]	2018	CH	2016*	Shanghai	≥60	PADL + IADL	4003	2257 (56.38)	NR	473	11.82	7
59.	Liu et al. [84]	2018	CH	2010–2014	Beijing	≥60 (70.3)	PADL	4499	2684 (59.66)	2397 (53.28)	544	12.10	8
60.	Xu et al. [85]	2018	CH	2016	Sichuan	≥60	PADL	890	577 (64.83)	NR	119	13.37	9
61.	Wu et al. [86]	2018	CH	2016*	Beijing	≥60	PADL + IADL	1158	713 (61.57)	NR	220	19.00	8
62.	Fu et al. [87]	2018	EN	2014	Hubei	≥65 (74.3)	PADL + IADL	1210	672 (55.54)	NR	249	20.58	7
63.	Liu et al. [88]	2018	EN	2016*	Hubei	≥65	PADL + IADL	622	358 (57.56)	NR	179	28.78	6
64.	Gu and Feng [89]	2018	EN	2000–2009	National	≥65 (88.1)	PADL **	32,281	18,914 (58.59)	NR	9361	29.00	9
65a.	Hou et al. [90]	2018	EN	1998	National	≥80 (92.0)	PADL	8768	5240 (59.76)	5455 (62.21)	3236	36.91	10
65b.	Hou et al. [90]	2018	EN	2000	National	≥80 (91.1)	PADL	10,940	6356 (58.10)	4181 (38.22)	3805	34.78	10
65c.	Hou et al. [90]	2018	EN	2002	National	≥80 (92.3)	PADL	10,905	6579 (60.33)	5785 (53.05)	4414	40.48	10
65d.	Hou et al. [90]	2018	EN	2005	National	≥80 (92.5)	PADL	10,393	6260 (60.23)	5723 (55.07)	3516	33.83	10
65e.	Hou et al. [90]	2018	EN	2008	National	≥80 (92.4)	PADL	11,658	7074 (60.68)	7016 (60.18)	3318	28.46	10
66.	Bai et al. [91]	2018	CH	2013	Hebei	≥60	PADL + IADL	1374	785 (57.13)	NR	584	42.50	7
67.	Gong et al. [92]	2018	EN	2016*	Anhui	≥60 (70.7)	PADL + IADL	3182	1862 (58.52)	3182 (100.00)	1942	61.03	6
68.	Dong et al. [93]	2018	EN	2014	Anhui	≥60	PADL + IADL	945	580 (61.38)	945 (100.00)	599	63.39	9
69a.	Zhang et al. [94]	2019	CH	2015	Beijing, Shanghai, Hebei, Sichuan, Yunnan, Guangxi	≥60	PADL	23,803	13,234 (55.60)	11,029 (46.33)	500	2.10	9
69b.	Zhang et al. [94]	2019	CH	2015	Beijing, Shanghai, Hebei, Sichuan, Yunnan, Guangxi	≥60	IADL	23,803	13,234 (55.60)	11,029 (46.33)	4570	19.20	9
79.	Li et al. [95]	2019	CH	2015	Fujian	≥60	PADL **	5174	2716 (52.49)	NR	280	5.41	9
71.	Liu et al. [96]	2019	CH	2016–2017	Hebei	≥60	PADL + IADL	3125	1670 (53.44)	NR	324	10.37	8
72.	Fu et al. [97]	2019	CH	2017 *	Sichuan	≥60	PADL **	1000	562 (56.20)	NR	158	15.80	7
73.	Chen et al. [98]	2019	EN	2016	Jiangsu	≥60	PADL	2493	1314 (52.71)	1584 (63.54)	402	16.13	7
74.	Chen et al. [99]	2019	EN	2014	National	≥80	PADL	4076	2308 (56.62)	2259 (55.42)	1083	26.57	9
75.	Xu et al. [100]	2019	CH	2017	Hunan	≥60	PADL + IADL **	194	NR	194 (100.00)	55	28.35	8
76.	Bai et al. [101]	2019	CH	2016–2017	Hebei	≥60	PADL + IADL **	6171	3024 (49.00)	NR	2489	40.33	9
77.	Ma et al. [102]	2019	CH	2016–2017	Hebei	≥60	PADL + IADL **	6171	NR	NR	2489	40.33	8
78.	Zhao et al. [103]	2019	CH	2017 *	Hebei	≥60 (75.5)	PADL + IADL	724	NR	NR	309	42.68	7
79.	Yao et al. [6]	2019	CH	2014–2016	Hainan	≥100 (102.8)	PADL	940	765 (81.38)	NR	670	71.28	9
80.	Chen et al. [104]	2020	CH	2015	National	≥60	PADL	4485	2422 (54.00)	NR	297	6.62	9
81.	Ning et al. [105]	2020	CH	2018	Shandong	≥60 (69.9)	PADL	3349	1715 (51.21)	NR	229	6.84	9
82.	Xu et al. [106]	2020	CH	2018 *	Hainan	≥60	PADL **	365	213 (58.36)	221 (60.55)	29	7.95	8
83.	Gu et al. [107]	2020	CH	2018	Jiangsu	≥60 (69.4)	PADL	3259	1644 (50.44)	1544 (47.38)	344	10.56	9
84.	Peng et al. [108]	2020	EN	2018	Guangdong	≥60 (71.6)	PADL	1321	NR	NR	160	12.11	8
85.	Xu et al. [20]	2020	EN	2018	Ningxia	≥60 (70.5)	PADL	1040	513 (49.33)	NR	179	17.21	8
86.	Zhang et al. [109]	2020	CH	2018*	Henan	≥60 (70.9)	PADL	5570	2825 (50.72)	4074 (73.14)	1139	20.45	6
87.	Cai et al. [110]	2020	CH	2015	Yunnan	≥60 (70.9)	PADL + IADL	3978	2213 (55.63)	2000 (50.28)	1017	25.57	9
88.	Song et al. [111]	2020	CH	2014	Shandong	≥65	PADL **	559	254 (45.44)	312 (55.81)	143	25.58	9
89.	Liu et al. [112]	2020	CH	2015–2018	Guangdong	≥60 (74.3)	PADL + IADL **	221	104 (47.06)	NR	58	26.24	9
90.	Du et al. [113]	2020	CH	2016	Anhui	≥60 (71.7)	PADL	983	527 (53.61)	NR	312	31.74	10
91.	Zhang et al. [114]	2020	CH	2016	Chongqing	≥65	PADL + IADL **	1341	609 (45.41)	NR	596	44.44	8
92.	Lin et al. [115]	2020	CH	2018 *	Yunnan	≥60 (76.7)	PADL	182	118 (64.84)	NR	96	52.75	8
93.	Xiao et al. [116]	2021	EN	2018	Guizhou, Yunnan, Sichuan, Xinjiang	≥60 (69.4)	PADL	3770	NR	NR	488	12.94	8
94.	Cheng and Yan [117]	2021	EN	1998–2014	National	≥80	PADL	30,317	17,663 (58.26)	NR	4884	16.11	10
95.	Gao et al. [118]	2021	CH	2017	Shandong	≥60 (69.8)	PADL + IADL	7070	4224 (59.75)	NR	1603	22.67	9
96.	Chen et al. [119]	2021	CH	2014	National	≥60 (70.5)	PADL	6182	3305 (53.46)	3337 (53.98)	1517	24.54	9
97.	Yan et al. [120]	2021	CH	2018	National	≥65 (85.6)	PADL	15,771	8902 (56.45)	NR	4196	26.61	10

* Survey year is the year the data were collected; if the survey year was not reported, the data was computed by subtracting two from the year of publication; ** Some studies did not indicate the source of the scale. We determined by the items and calculation methods used; CH = Chinese; EN = English; NR = not reported.
